# Supplementary material for: Unveiling genome plasticity and a novel phage in Mycoplasma felis: Genomic investigations of four feline isolates
Source: Microb Genom. 2024 Mar 28;10(3):001227. doi: 10.1099/mgen.0.001227 (PMC11004492; doi:10.1099/mgen.0.001227)
Supplement: Uncited Supplementary Material 1. [file mgen-10-01227-s001.pdf]

Supplementary Data

Supplementary Table 1. Summary of DNA sequencing results after trimming and quality filtering

	MF047	MF219	MF329	MF632
**Illumina reads**				
Total paired reads	8,977,712	6,647,214	6,481,506	6,807,888
Median length	151	151	151	151
>Q20 (%)	96.93	95.7	96.83	96.99
>Q30 (%)	91.31	88.43	91.12	91.41
GC (%)	24.64	24.67	26.35	24.85
**Nanopore reads**‡				
Total reads	65,383	9,515	103,977	19,128
Median length	7,049	6,563	3,717	5,058.5
N50	16,885	22,105	12,331	16,399
>Q20 (%)	48.38	51.69	41.64	49.27
>Q30 (%)	15.12	16.77	11.64	15.74
GC (%)	24.9	24.79	27.19	25.08

‡Results presented are from combining two nanopore sequencing runs

Supplementary Table 2. Summary of assemblies and annotations of four isolates from this study, compared to the complete reference sequence for *Mycoplasma felis* (Myco-2)

Isolate	Myco-2	MF047	MF219	MF329	MF632
**Accession**	NZAP022325	CP114890	CP114889	CP115656	CP114888
**Assembly information**					
Length (bp)	841,695	948,716	945,056	936,813	905,741
CheckM completeness (%)	99.21	99.21	99.21	99.21	99.21
PGAP ANI*	98.4%	98.5%	98.6%	98.6%	98.5%
**Median sequencing depth**					
Short reads	-	1762	1372	467	1499
Long reads	-	695	110	88	181
**Genomic features**					
CDS	740	759	777	759	743
ncRNA	2	2	2	2	2
rRNA	9	9	9	9	9
Regulatory	2	2	2	2	2
tRNA	29	30	29	29	30
tmRNA	1	1	1	1	1
**Genes of interest**					
Secretory Systems	0	2	1	3	1
Transposase	22	40	52	27	38

* average nucleotide identity to *Mycoplasma felis* ATCC 23991 according to the internal PGAP ANI taxonomic classification

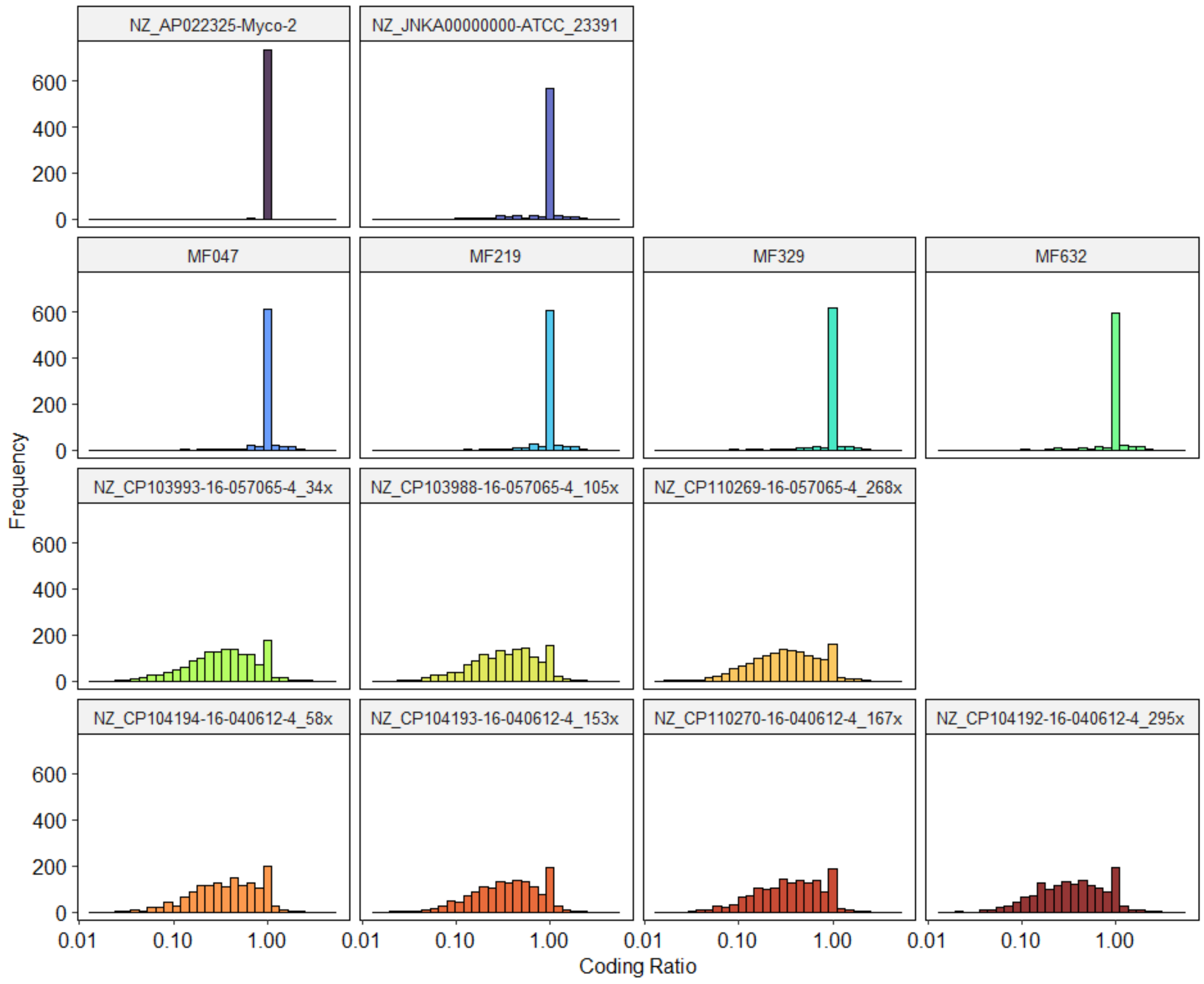

Supplementary Figure 1. Coding ratio frequency counts across the *Mycoplasma felis* genomes from this study and those currently available on GenBank. Coding ratios were determined by dividing the amino acid sequence length of annotated proteins by the best hit match in a custom UniProt Mycoplasma database. The coding ratio axis is log transformed for visualisation. Title names for NCBI origin data include the accession number and strain name (e.g. NZ_CP104194, and 16-040612-4). For Oxford Nanopore data from NCBI, the depth of coverage is added from NCBI (e.g. 58 x).

Supplementary Table 3. Average nucleotide identities (ANI) of Australian *M. felis* isolate genomes, the genome of the equid *M. felis* reference strain (Myco-2) and felid *M. felis* contig datasets (Genbank accessions: MF047: CP114890; MF219: CP114889; MF329: CP115656; MF632: CP114888; Myco-2: AP022325; 16-057065-4: CP103988; 16-040612-4:CP104192; ATCC23391: JNKA01000000).

	MF047	MF219	MF329	MF632	Myco-2	16-057065-4	16-040612-4
**MF219**	99.79	-	-	-	-	-	-
**MF329**	98.21	98.16	-	-	-	-	-
**MF632**	98.21	98.17	98.33	-	-	-	-
**Myco-2**	98.14	98.06	98.18	98.17	-	-	-
**16-057065-4**	97.77	97.80	98.25	97.77	97.77	-	-
**16-040612-4**	97.65	97.65	97.84	97.80	97.72	97.65	-
**ATCC23391**	98.31	98.29	98.39	98.27	98.33	97.95	97.95

Supplementary Table 7. Phage ORF table excluding hypothetical proteins. BLASTx amino acid pairwise identity is shown for conserved coding sequences found within other mycoplasma species.

Min	Name	Length	*M. mustelae*	*M. molare*	*M. agalactiae*	*M. bovis*
1293	HNH endonuclease	528	41%	57%	56%	53%
2987	Metallo-hydrolase	807	38%	40%	32%	33%
3775	Endonuclease	357	51%	51%	47%	47%
4303	DNA Helicase	1368	53%	49%	48%	48%
7406	Methionine adenosyltransferase	1209	-	42%	-	-
8607	DNA cytosine methyltransferase	1554	34%	34%	35%	-
10172	DNA polymerase	2010	59%	52%	49%	49%
12194	DNA Primase	2016	54%	48%	42%	42%
14231	Xer Recombinase	750	44%	36%	35%	36%
24454	Bacteriophage Gp15	243	44%	44%	42%	40%
27470	Phage prohead protein	1872	64%	54%	45%	52%
29331	Phage portal protein	1254	69%	67%	60%	60%
30695	Terminase	1800	62%	60%	58%	58%
